# Correlation between magnetic resonance imaging proton density fat fraction (MRI-PDFF) and liver biopsy to assess hepatic steatosis in obesity

**DOI:** 10.1038/s41598-024-57324-3

**Published:** 2024-03-22

**Authors:** Pornphan Wibulpolprasert, Benya Subpinyo, Supphamat Chirnaksorn, Prapimporn Chattranukulchai Shantavasinkul, Supanee Putadechakum, Sith Phongkitkarun, Chanika Sritara, Napat Angkathunyakul, Preeda Sumritpradit

**Affiliations:** 1 Department of Diagnostic and Therapeutic Radiology, Mahidol University, Bangkok, 10400 Thailand; 2 Department of Medicine, Mahidol University, Bangkok, 10400 Thailand; 3 Department of Pathology, Mahidol University, Bangkok, 10400 Thailand; 4 Department of Surgery, Faculty of Medicine, Ramathibodi Hospital, Mahidol University, Bangkok, 10400 Thailand

**Keywords:** Diseases, Medical research

## Abstract

Obesity is highly associated with Non-alcoholic fatty liver disease (NAFLD) and increased risk of liver cirrhosis and liver cancer-related death. We determined the diagnostic performance of the complex-based chemical shift technique MRI-PDFF for quantifying liver fat and its correlation with histopathologic findings in an obese population within 24 h before bariatric surgery. This was a prospective, cross-sectional, Institutional Review Board-approved study of PDFF-MRI of the liver and MRI-DIXON image volume before bariatric surgery. Liver tissues were obtained during bariatric surgery. The prevalence of NAFLD in the investigated cohort was as high as 94%. Histologic hepatic steatosis grades 0, 1, 2, and 3 were observed in 3 (6%), 25 (50%), 14 (28%), and 8 (16%) of 50 obese patients, respectively. The mean percentages of MRI-PDFF from the anterior and posterior right hepatic lobe and left lobe vs. isolate left hepatic lobe were 15.6% (standard deviation [SD], 9.28%) vs. 16.29% (SD, 9.25%). There was a strong correlation between the percentage of steatotic hepatocytes and MRI-PDFF in the left hepatic lobe (r = 0.82, *p* < 0.001) and the mean value (r = 0.78, *p* < 0.001). There was a strong correlation between MRI-derived subcutaneous adipose tissue volume and total body fat mass by dual-energy X-ray absorptiometry, especially at the L2–3 and L4 level (r = 0.85, *p* < 0.001). MRI-PDFF showed good performance in assessing hepatic steatosis and was an excellent noninvasive technique for monitoring hepatic steatosis in an obese population.

## Introduction

The global prevalence of overweight and obesity has dramatically increased over the past four decades. In 2016, about 2 billion adults were overweight. Of these, over 650 million adults worldwide were obese^1^. This has increased the mortality risk and significant medical and psychological comorbidities. Obesity and insulin resistance have a primary pathophysiological role in the development of non-alcoholic fatty liver disease (NAFLD)^2^, which affects approximately 10–24% of the general population and 57–74% of obese persons^3^.

Hepatic steatosis is defined as the excessive accumulation of triglycerides in hepatocytes^4^. NAFLD refers to a broad spectrum of liver damage from simple steatosis to steatohepatitis and cirrhosis^3^. Previous studies showed that about 30% of patients with NAFLD had progression to liver damage, and 40% suffered from liver-related morbidity and mortality^4–6^. Weight loss is recommended for all overweight patients and can significantly improve steatohepatitis and fibrosis^7,8^. Bariatric surgery is the most effective surgical procedure to promote long-term weight loss and resolve obesity-related comorbidities, including NAFLD, in morbidly obese subjects^9^.

A diagnosis of NAFLD by clinical manifestations or liver function has poor predictive value, and liver biopsy is the gold standard for determining the severity and confirming a diagnosis of NAFLD. However, limitations of liver biopsy include interference, the potential for severe complications, and subjective sample variability. Noninvasive imaging tools have been used to qualify and quantify hepatic fat content, including conventional B-mode^10,11^ and quantitative ultrasound techniques by measuring various acoustic parameters such as elastography, attenuation coefficient, and controlled attenuation parameter^12^, measurement of hepatic attenuation in conventional computer tomography (CT)^13–16^, advanced CT-based methods (e.g., single-energy quantitative CT [QCT], dual-energy CT [DECT], and photon-counting CT)^17–19^, and MRI-based techniques (MR spectroscopy and muti-echo Dixon MRI)^20^. However, there were some limitations due to low sensitivity for early detection, standardized image acquisition and analysis for quantitative hepatic fat content by ultrasound, and poor image quality due to skin and subcutaneous fat thickness in obese patients^21,22^. Furthermore, the ionizing radiation exposure from CT and the complexity of MR spectroscopy techniques were the significant limitations to widespread use^23,24^.

Several MR image-based techniques have been used for fat detection and quantifying the hepatic fat and water content, including the Dixon method (in-phase and out-of-phase images), frequency-selective imaging, and MR spectroscopy^20,25^. Applying a 3D multi-echo chemical-shift encoded technique, PDFF can be calculated for the entire liver. This technique considers for essential confounders as magnetic field distribution, T1-relaxation and spectral complexity of fat by implementing a low flip angle, seven-peak fat modeling and T2* correction. The proton density fat fraction (PDFF) calculation is a chemical shift-based water and fat signal separation technique, which can cover the entire liver in a single breath-hold^23,26–28^.

Several studies have shown the high performance of MRI-PDFF for liver fat quantification in variable study cohorts and the longer duration gap between MRI and the reference tests that may affect the change of hepatic fat content over time. Therefore, this study aimed to evaluate the correlation between liver fat quantification by MRI-PDFF and histopathologic findings in an obese population who underwent bariatric surgery within 24 h of MRI and the biopsy interval gap. Furthermore, the correlation of MRI-derived visceral adipose tissue (VAT) and subcutaneous adipose tissue (SAT) volume measurements with the percentage of histologic analysis was assessed in this study.

## Methods

### Study design and patient population

The human research ethics committee, Faculty of Medicine Ramathibodi Hospital, Mahidol University, approved this prospective data collection study and followed all ethical rules. All methods were performed in accordance with the Declaration of Helsinki. Subjects were enrolled at a bariatric surgery clinic, Ramathibodi Hospital, Mahidol University, Thailand. They consisted of obese patients undergoing bariatric surgery (Roux-en-Y gastric bypass and sleeve gastrectomy) at Ramathibodi Hospital between December 2018 and February 2022. All subjects were informed about the study protocol and provided informed consent. Standardized clinical evaluation was performed, and laboratory data were collected. Obese adults (≥ 18 years old) who have body mass index (BMI) greater or equal to 30 kg/m^2^) were enrolled if they were willing and able to provide informed consent and able to undergo pre-operative MRI before liver biopsy during bariatric surgery within the next 24 h. Patients were excluded if their body weight was over 160 kg (due to limitation of MRI aperture diameter), they could not provide informed written consent, or they were pregnant.

### MRI examination

Patients were examined supine with a standard torso phased-array centered over the liver within a 3.0-Tesla MR machine (Ingenia; Philips Healthcare, Best, Netherlands). Multi-echo DIXON sequence axially covering the entire liver in a single breath-hold, covering the whole liver in the axial plane, anatomic imaging of the upper abdomen with axial T2-weighted fat saturation, as well as multi-echo DIXON image volume centered on the umbilical, L2–L3, and L4 levels in three planes (axial, coronal, and sagittal planes).

#### PDFF mapping

**Figure 1:**
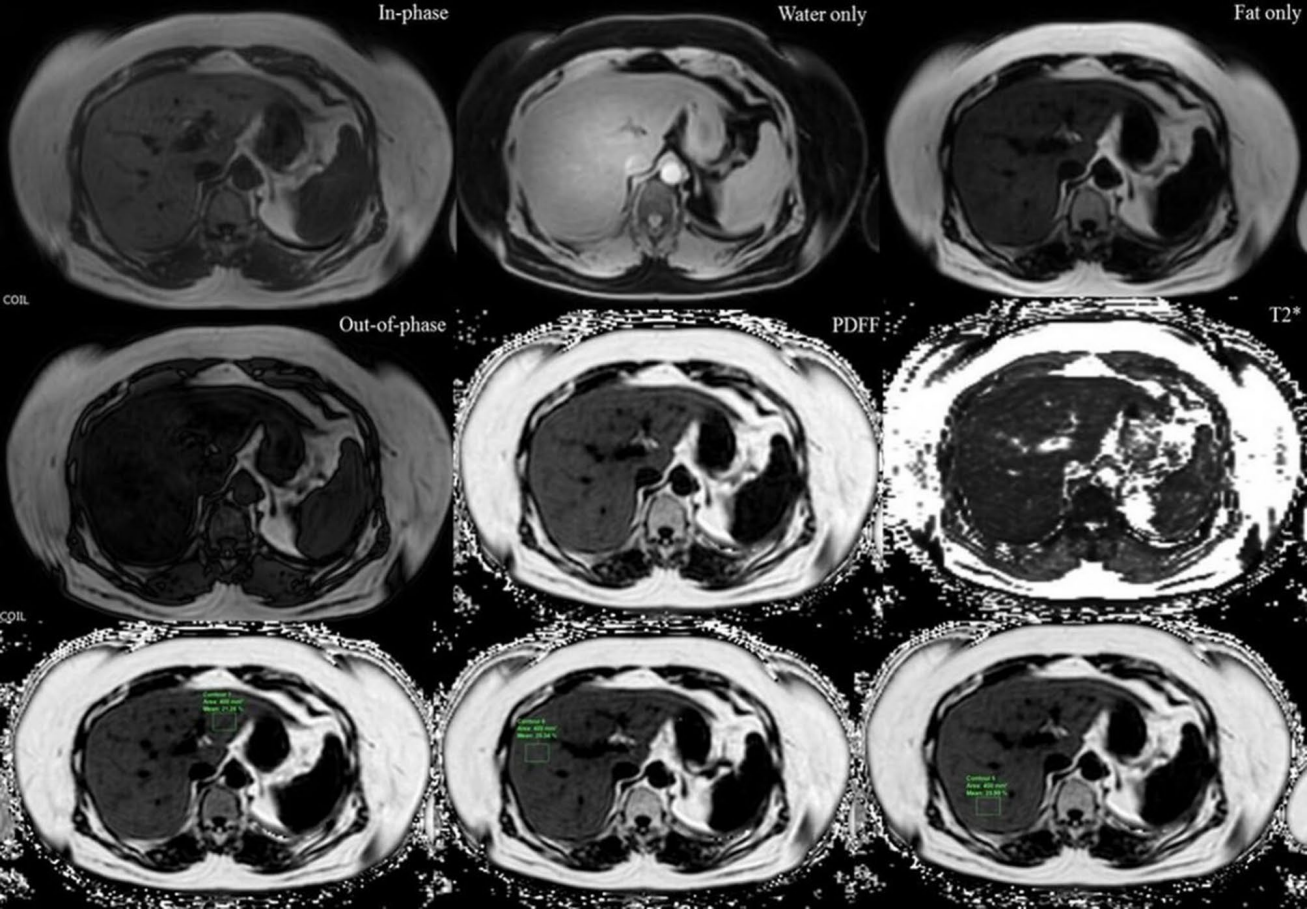
Moderate hepatic steatosis (40% steatosis on histologic analysis and 21.5% of PDFF) in a 63-year-old female on multi-echo DIXON image and PDFF map image. On the PDFF images (third row), mean percentage of PDFF of the left hepatic lobe 21.3%, anterior right hepatic lobe 25.3% and posterior right hepatic lobe 26%.

The multi-echo sequence automatically produces water, fat, fat fraction, R2*, and T2* maps (Fig. 1). Then, the PDFF maps were corrected for confounding factors, including phase error correction and T2* effects. The muti-echo DIXON MRI protocol included the following parameters: six TEs with first TE of 0.91 ms and delta TE of 0.7 ms; TR of 5.3 ms; flip angle (FA) of 3°; field of view (FOV) of 500 × 500 mm; the matrix size of 168 × 168; slice thickness of 3 mm with 60 image slices: acquisition time of 12 s.

#### Subcutaneous and visceral adipose tissue volume (SAT and VAT) imaging

A multi-echo DIXON scan was acquired to cover the diaphragm and symphysis pubis. In phase, opposed phase, water, and fat-only images were separated on the scanner using the mDIXON algorithm. The imaging parameters for the multi-echo DIXON image volume scan were two TEs (1.04 ms and 2.0 ms), TR of 3.3 ms, and FA of 3° with 10 image slices. The slice thickness was 5 mm, the FOV was 500 × 500 mm, and the matrix size was 168 × 168. The acquisition times for multi-echo axial, sagittal, and coronal planes were 15 s, 12 s, and 12 s, respectively.

### Imaging analysis

MR images of all cases were loaded into the picture archiving and communications system (PACS) at Ramathibodi Hospital, using DICOM Conformance (Synapse version 3.2.0, Fujifilm Medical Systems USA Synapse^®^ PACS System, USA). All pre-operative MR images were reviewed, and a consensus was reached by a radiologist and last year's resident trainee, blinded to the histopathologic results.

#### PDFF analysis

There is no consensus on a standardized approach to measuring liver fat with manually drawn regions of interest^29^. Although the placement of largest-fit-possible ROIs in all nine Couinaud hepatic segments was shown to be the most reproducible and repeatable method^30^ due to the heterogeneous pattern of hepatic steatosis, it is time-consuming and a limitation for clinical practice workflow. For reasonable time burden compromised and reproducibility concern with acceptable limits of agreements^31^, three ROIs of 4 cm^2^ were carefully placed on the PDFF map images by devoiding the large vessels, large bile ducts, organ boundaries, focal hepatic lesion, and imaging artifacts. Three ROIs per subject were obtained from the left hepatic lobe and anterior and posterior segments of the right hepatic lobe (Fig. 1). The mean PDFF of the liver was calculated manually from three ROIs. Then, the mean PDFF in each of the three ROIs and the mean PDFF of the liver were recorded.

#### Subcutaneous and visceral adipose tissue (SAT and VAT) volume measurements

**Figure 2:**
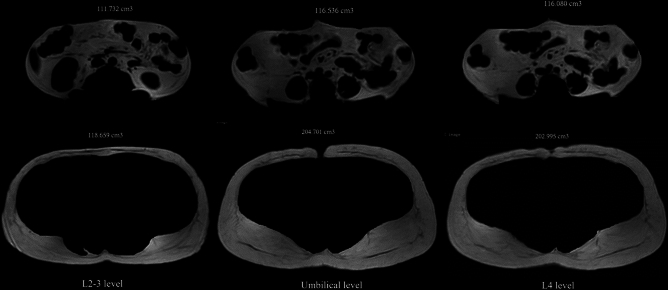
Obesity with BMI 36.4 kg/m^2^. Visceral adipose tissue volumes of L2-3, umbilical, and L4 levels (top row, from left to right). Subcutaneous adipose tissue volumes of L2-3, umbilical, and L4 levels (bottom row, from left to right).

The differentiation of abdominal tissue compartments was based on the water and fat separation images of the multi-echo DIXON scan. The visceral and subcutaneous adipose tissue volumes were identified using a pixel density plot across a fat-soft tissue boundary and selecting the signal intensity value. The SAT and VAT compartments were manually bound using Philips Interspace Portal (Philips Healthcare). Measurements of SAT and VAT were taken from two contiguous MRI slides of 5-mm-thickness of the axial image at the umbilical, L2–L3, and L4 levels (Fig. 2).

### Transient elastography

Hepatic steatosis was assessed using the controlled attenuation parameter (CAP) values in dB/m provided by the FibroScan^®^502 Touch model (XL Probe; Echosens, Paris, France) by a trained technician. The CAP measurement derived from transient elastography within 180-day intervals from liver biopsy, including 45 patients, was analyzed. The median time interval and interquartile range (IQR) were 40 days (IQR, 24–77 days).

### Dual energy X-ray absorptiometry (DXA)

The body composition was evaluated by a GE LUNAR iDXA Narrow-Angle Dual Energy X-ray Densitometer (GE Healthcare, Madison, WI). The patients were scanned using standard imaging and positioning protocols. The encore software automatically determined the ROIs for the total body, arms, legs, trunk, android, and gynoid regions. The total body fat measurement derived from DXA within 180-day intervals from liver biopsy, including 42 patients, was analyzed. The median time interval and interquartile range (IQR) were 36 days (IQR, 19–54 days).

### Liver biopsy

**Figure 3:**
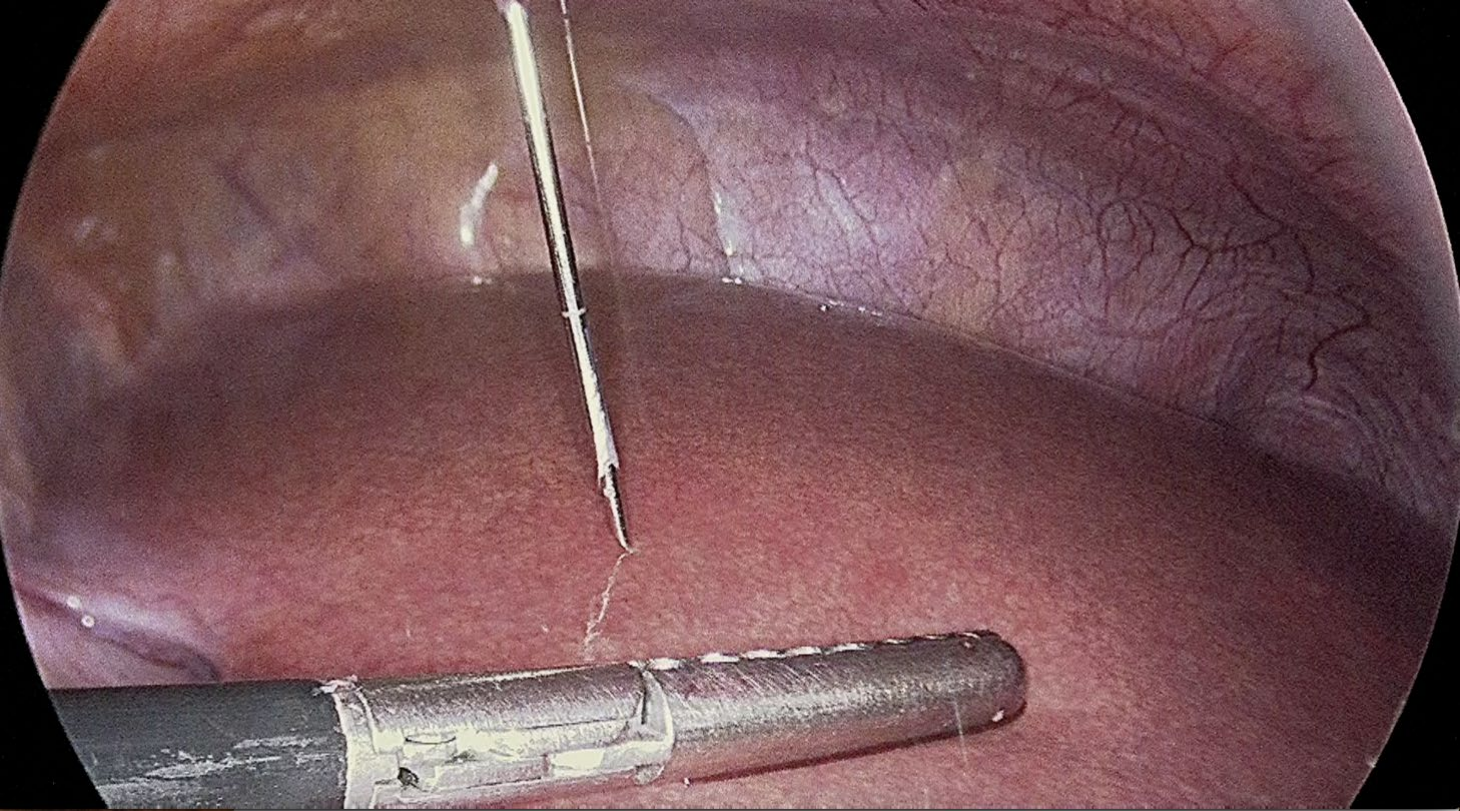
True-cut needle biopsy of the liver at Couinaud hepatic segments III during bariatric surgery.

After general anesthesia and using sterile techniques, the camera port was inserted, and a pneumoperitoneum was created. The same surgeon performed an 18-gauge laparoscopic-guided Tru-cut needle biopsy of the left hepatic lobe (Couinaud hepatic segments III) during bariatric surgery (Fig. 3). One liver specimen was obtained from each patient. One biopsy was performed per patient.

### Histopathologic analysis

**Figure 4:**
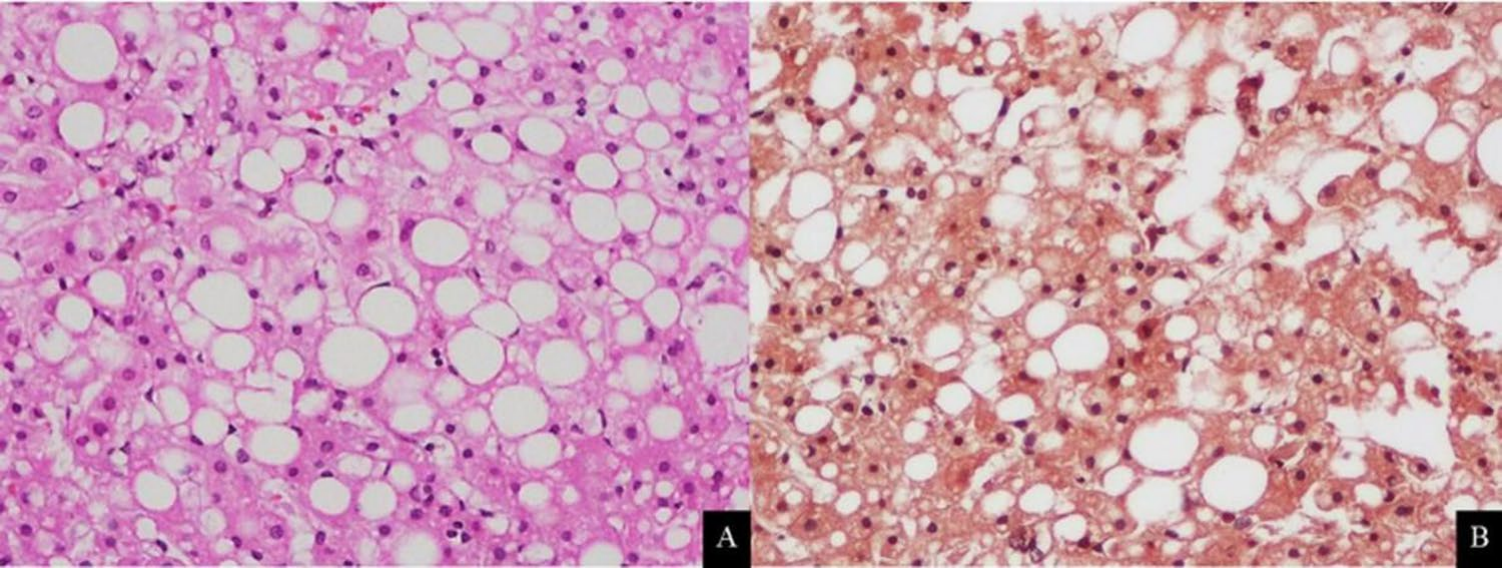
Moderate hepatic steatosis in a 63-year-old female. H&E staining of liver parenchyma (**A**) reveals 40% of mixed macro- and microvesicular steatosis. Masson trichrome staining of liver parenchymal (**B**) shows no perisinusoidal fibrosis.

Liver biopsy specimens were fixed in 10% formalin and stained with hematoxylin–eosin (H&E) and Masson's trichrome (Fig. 4). All biopsy specimens were interpreted by the same expert hepatopathologist, blinded to the clinical and biological data. The histopathologic findings were classified using the NASH Clinical Research Network Scoring System^16^.

### Statistical analysis

Pearson's correlation coefficient was used to evaluate the degree of association between the percentage of MRI-PDFF and the percentage of steatotic hepatocytes from histopathologic analysis, as well as the degree of association between MRI-derived SAT or VAT and the body mass index. The ROC curve was used to calculate the diagnostic performance of MRI-PDFF. Statistical significance was considered when *p* values < 0.05. The cut-off value of MRI-PDFF for dichotomized steatosis grade was determined based on the best area under the ROC curve analysis.

## Results

### Patients

Seventy-five patients were candidates for bariatric surgery between December 2018 and February 2022. Twenty-five patients were excluded because they were overweight (> 160 kg; n = 6), did not agree to participate in the study after informed consent (n = 14), and had contraindications for MRI scanning, especially claustrophobia (n = 5). Thus, the study included 50 patients with MRI imaging and liver biopsy. Their physical characteristics are shown in Table 1. There were 15 men (30%) and 35 women (70%). The mean age was 38 years (standard deviation [SD], 9.1 years), the mean body weight was 118.3 kg (SD, 20 kg), and the mean BMI was 44.1 kg/m^2^ (SD, 6.52 kg/m^2^). DXA's mean total body fat mass was 57.70 kg (SD, 13.21 kg). The mean CAP using transient elastography was 335.93 dB/m (SD, 44.92).

**Table 1:** Demographic characteristics of 50 patients.

Patient characteristics	Result (n = 50)
Gender: number (%)	
Male	15 (30)
Female	35 (70)
Age (year); mean (SD.)	38 (9.1)
Body weight (kg); mean (SD.)	118.3 (20)
BMI (kg/m^2^); mean (SD)	44.1 (6.52)
Total body fat mass by DXA(kg); mean (SD) (n = 42)	57.70 (13.21)
Underlying disease; number (%)	
Diabetes mellitus	31 (62)
Hypertension	23 (46)
Dyslipidemia	37 (74)
Stroke	2 (4)
Obstructive sleep apnea	43 (86)
Vitamin D deficiency	47 (94)
CAP (dB/m); mean (SD) (n = 45)	335.93 (44.92)

### Histopathologic findings

The histopathologic results (Table 2) showed that the median percentage of steatotic hepatocytes was 30% (0–90%). Hepatic steatosis was graded as follows: 6% (3/50) were grade 0, 50% (25/50) were grade 1, 28% (14/50) were grade 2, and 16% (8/50) were grade 3. Lobular inflammation was graded as follows: 56% (28/50) were grade 0, 42% (21/50) were grade 1, and 2% (1/50) were grade 2. Hepatocellular ballooning was graded as follows: 34% (17/50) were grade 0, 56% (28/50) were grade 1, and 10% (5/50) were grade 2. Fibrosis was graded as follows: 74% (37/50) were grade 0, and 26% (13/50) were grade 1.

**Table 2:** Histopathologic results.

Histopathological results	Number (%)
Hepatic steatosis	
Grade 0; < 5% hepatocytes	3 (6%)
Grade 1; 5–33% hepatocytes	25 (50%)
Grade 2; 34–66% hepatocytes	14 (28%)
Grade 3; > 66% hepatocytes	8 (16%)
*Median percentage of steatotic hepatocytes (range)	30 (0–90%)
Lobular inflammation	
Grade 0; no foci	28 (56%)
Grade 1; < 2 foci per 200Xfield	21 (42%)
Grade 2; 2–4 foci per 200Xfield	1 (2%)
Grade 3; > 4 foci per 200Xfield	0 (0%)
Hepatocellular ballooning	
Grade 0; none	17 (34%)
Grade 1; few cells	28 (56%)
Grade 2; many cells	5 (10%)
Fibrosis	
Grade 0; none	37 (74%)
Grade 1; perisinusoidal or periportal	13 (26%)
Grade 2; perisinusoidal and periportal	0 (0%)
Grade 3; bridging fibrosis	0 (0%)
Grade 4; cirrhosis	0 (0%)

### MRI-PDFF

The mean MRI-PDFF percentage of the left hepatic lobe and mean or average value from both hepatic lobes were 15.6% (SD, 9.28%) and 16.3% (SD., 9.25%), respectively. The mean MRI-PDFF of the left hepatic lobe region and mean values from three ROIs (right anterior, right posterior, and left hepatic lobes) were 3.9% (SD, 1.2%) and 3.1% (SD, 1.4%) in patients with steatotic grade 0 (3/50), 10.4% (SD, 6.3%) and 11.4% (SD, 6.8%) in patients with steatotic grade 1 (25/50), 21.2% (SD, 7%) and 22% (SD, 6.6%) with steatotic grade 2 (14/50), and 26.8% (SD, 4%) and 26.5% (3.9% ) in those with steatotic grade 3 (8/50), respectively.

### MRI-derived SAT and VAT volume measurements

The mean subcutaneous adipose tissue volume measurements at L2–3, umbilical, and L4 levels were 235.6 cm^3^ (SD, 86.9 cm3), 296.3 cm^3^ (SD, 101.96 cm^3^), and 297.4 cm^3^ (SD, 93.42 cm^3^), respectively. The mean visceral adipose tissue volumes at L2–3, umbilical, and L4 levels were 116.2 cm^3^ (SD, 42.97 cm^3^), 104.2 cm^3^ (SD, 36.42 cm^3^), and 109.7 cm^3^ (SD, 37.54 cm^3^), respectively.

### Correlation analysis (dichotomized steatosis grades)

There was a strong correlation between the percentage of MRI-PDFF measured from the left hepatic lobe and the mean from the left and right hepatic lobes with the percentage of steatotic hepatocytes by histologic analysis with correlation coefficients of (r) 0.82 (*p* value, < 0.001) and of 0.78, respectively (*p* value, < 0.001) (Table 3).

**Table 3:** MR imaging results.

MR imaging findings	Result (n = 50)	Pearson's correlation coefficient (r) with the percentage of steatotic hepatocytes by histologic analysis	*p* Value*
Mean percentage of MRI-PDFF (SD.)			
Left hepatic lobe	15.6 (9.28)	0.82	< 0.001*
Average of the liver	16.3 (9.25)	0.78	< 0.001*
Mean subcutaneous adipose tissue volume (cm^3^) (SD.)			
L2-3 level	235.6 (86.9)	0.38	0.007*
Umbilical level	296.3 (101.9)6	0.34	0.02*
L4 level	297.4 (93.42)	0.33	0.20
Mean Visceral adipose tissue volume (cm^3^) (SD.)			
L2-3 level	116.2 (42.97)	0.14	0.32
Umbilical level	104.2 (36.42)	0.26	0.07
L4 level	109.7 (37.54)	0.13	0.38

*Statistical significance was considered when *p* values < 0.05.

In this study, to distinguish patients with steatosis grade 0 (3/50) and grade 1(25/50) from those with grade 2 or greater (22/50), a 16.8% MRI-PDFF threshold provided 90.9% sensitivity, 85.7% specificity, 83.3% positive predictive value (PPV), and 92.3% negative predictive value (NPV) with the best area under the ROC of 0.88 (95% confidence interval [CI] 0.79–0.97). This threshold was in the range of various outsets from previous studies validated earlier, ranging from 11.3 to 17.5%, with summary sensitivity and specificity of 0.86–0.96 and 0.68–0.86, respectively (Table 4).

**Table 4:** Diagnostic performance of MRI-PDFF and CAP of this study for the diagnosis of steatosis grade 0–1 versus 2–3 by different previously published thresholds.

Author and year of references	Threshold (References)	Number of the reference population	Sensitivity, %	Specificity, %	PPV, %	NPV, %	AUROC (95% CI)	*p* Value*
MRI-PDFF								0.316
This study	16.8% (this study)	50	90.9	85.7	83.3	92.3	0.88 (0.79–0.97)	
Tang et al.^28^	17.4%	77	86.4	85.7	82.6	88.9	0.86 (0.76–0.96)	
Tang et al.^35^	16.4%	89	90.9	85.7	83.3	92.3	0.88 (0.79–0.97)	
Imajo et al.^33^	11.3%	142	95.5	67.9	70.2	95.0	0.82 (0.72–0.92)	
Park et al.^36^	13.0%	103	95.5	71.4	72.4	95.2	0.83 (0.74–0.93)	
Middleton et al.^37^	16.3%	113	90.9	85.7	83.3	92.3	0.88 (0.79–0.97)	
Middleton et al.^38^	17.5%	169 (children)	86.4	85.7	82.6	88.9	0.86 (0.76–0.96)	
CAP in Castera et al.^32^	268 dB/m*	2735 patients (537 with NAFLD; 19.6%)	95.2	12.5	48.8	75	0.54 (0.46–0.62)	< 0.001
CAP this study	342 dB/m	50	66.7	66.7	63.6	69.6	0.70 (0.54–0.85)	

*Statistical significance was considered when *p* values < 0.05.

The performance of CAP measurements to differentiate moderate or severe steatosis from mild or no hepatic steatosis in this study was determined using a threshold of 342 dB/m with the best area under the ROC of 0.70 (95% confidence interval [CI] 0.54–0.85). This threshold provided 66.7% sensitivity, 66.7% specificity, PPV 63.6%, and NPV 69.6%. When using the threshold of 268 dB/m reported by Castera et al.^32^, the sensitivity, specificity, PPV, and NPV of our study were 95.2%, 12.5%, 48.8%, and 75% with an area under the ROC (AUROC) of 0.54 (95% CI 0.46–0.62) (Table 4).

There was a strong correlation between MRI-derived SAT volumes at L2–3, umbilicus, and L4 levels and the BMI, with correlation coefficients (r) of 0.77 (*p* value < 0.001), 0.69 (*p* value < 0.001), and 0.76 (*p* value < 0.001), respectively. The study also showed a good correlation between the SAT volume at L2–3, umbilicus, and L4 levels and total body fat mass by DXA (r = 0.85, r = 0.78, and r = 0.85 with *p* values of < 0.001, respectively). There was a weak correlation between the VAT volumes at L2–3 (r = 0.13, *p* value = 0.40), umbilical (r = 0.12, *p* value = 0.47), and L4 (r = 0.14, *p* value < 0.001) levels and total body fat mass by DXA. A weak correlation was noted between the visceral or subcutaneous adipose tissue volume and the percentage of steatotic hepatocytes by histologic analysis (r = 0.13–0.38, *p* value = 0.07–0.38).

## Discussion

The results of the current study support previous findings regarding the strong correlation between the percentage of MRI-PDFF and steatotic hepatocytes assessed by histologic analysis^5,28,33,34^. Our results support using a cut-off point of MRI-PDFF to differentiate liver fat content. No specific biochemical or serologic tests are competent for detecting and grading hepatic steatosis. Our results confirmed that the percentage of MRI-PDFF was strongly correlated with the relative amount of steatotic hepatocytes. Therefore, the MRI-PDFF is a essential calculated method from non invasive imaging techniques for steatosis grading and monitoring after treatment. However, it should be noted that MRI-PDFF determines the proportion of protons contained within fat molecules compared with all visual MRI signals. In contrast, histologic analysis measures the fraction of hepatocytes that contain macrovesicles of fat.

The various MRI-PDFF thresholds used to distinguish steatosis grade ≤ 1 from grade ≥ 2 steatoses were validated previously in extensive pilot studies^28,33,35–38^ (Table 4). The validated thresholds were ranging from 11.3 to 17.5%. There were slight differences between these validated thresholds, probably because of the different steatotic grades in diverse background liver populations and different MRI-to-biopsy interval gaps.

However, our study revealed no statistically significant differences in the diagnostic performance of MRI-PDFF for dichotomized liver steatosis grades 0/1 and ≥ grade 2 when using the previously published thresholds with an AUROC of 0.82–0.88. Furthermore, this study showed the best performance with an outset of 16.8%, which is in the range of various thresholds from previous studies and close to the initial analysis of Tang et al.^35^ This study population consisted of obese patients who were candidates for bariatric surgery and who had a higher risk of NAFLD and of suffering from liver-related complications. MRI-PDFF was an excellent calculation method derived from noninvasive imaging techniques for monitoring hepatic steatosis in an obese population. Furthermore, Tang et al.^28^ and Idilman et al.^5^ reported that the hepatic fat content might change over time. They found a high correlation between MRI-PDFF and histologic results using incrementally narrowed MRI-biopsy time intervals. Therefore, using a very narrow MRI-biopsy time interval compared with previous studies emphasized the precise diagnostic performance of MRI-PDFF in this study.

MRI-PDFF was significantly more accurate than transient elastography (*p* < 0.001), corresponding with previous studies^33,36^. Our results were similar to previous studies showing that MRI-PDFF was more precise for hepatic steatosis than CAP, which had high sensitivity (95.2%) but low specificity (12.5%) for discriminating hepatic steatosis greater than 33% (≥ grade 2) when using validated threshold by Castera et al.^32^ (268 dB/m) and moderate sensitivity (66.7%) and specificity (66.7%) when using the calculated threshold of this study (342 dB/m). The different performance on different thresholds of CAP is probably due to the different steatosis prevalence of the studies, the different durations between CAP and reference tests and different typs of CAP probes. Furthermore, accurate transient elastography depends on the operator’s expertise and other patient factors, including the BMI, intercostal space width, amount of intraperitoneal fluid, and visceral fat volume. These factors may cause limitations in CAP, especially in the obese population. Accordingly, MRI-PDFF may be more desirable for accurate steatosis quantification.

Although the BMI is widely used to determine adiposity and is easy to calculate, it was imprecise when differentiating body compositions, especially fatty tissues and muscle mass. Meeuwsen et al.^24^ reported a weak association between BMI in 18–25 and 25–32 kg/m^2^ and body fat, with a correlation of 0.21 and 0.38 in males and 0.38 and 0.40 in females, respectively. Therefore, body fat mass by DXA might be a more accurate and reliable method for monitoring total body fat change in an obese patient before and after bariatric surgery than simplified BMI. This study found a strong correlation between the SAT volume and total body fat mass by DXA. The highest correlation was at the L2–3 and L4 levels (r = 0.85), followed by the umbilical level (r = 0.78). These results suggest the subcutaneous adipose tissue volume at the L2–3 or L4 level was optimal for baseline subcutaneous adipose tissue volume measurements before bariatric surgery. These results correspond with those of previous studies,^39,40^. They suggest that a single intra-abdominal fat area at L2–3 intervertebral disc levels is preferable when determining intra-abdominal fat volumes. In contrast, the abdominal VAT volume was independent of general obesity. The correlation coefficient (r) values for the bivariate associations of the visceral adipose tissue at the L2–3, umbilical, and L4 levels with total body fat mass ranged from 0.12 to 0.14.

Although MRI-PDFF is emerging as a valid biomarker for steatosis, it does not ultimately provide many histologic features of NAFLD, especially the degree of necroinflammatory activity and stage of fibrosis. Assessing these histologic endpoints will require developing and validating other noninvasive quantitative imaging biomarkers. This study's strength included using a well-characterized, prospective cohort of NAFLD patients who were candidates for bariatric surgery and undergoing a liver biopsy within a 24-h interval between MRI-PDFF and liver biopsy and an excellent multidisciplinary team approach in a single institute among the pandemic COVID-19 era obstacles.

This study had some limitations. First, this study had a small sample size and lacked the power to detect differences in stage-by-stage changes in liver fat content. Second, drawing the ROIs on the MRI-PDFF image was not fully standardized. We measured one ROI for each location. Third, patient selection bias might have affected the results of the liver biopsies, which are more likely to be performed on NAFLD patients.

In conclusion, MRI-PDFF performed well in assessing hepatic steatosis and was an excellent noninvasive technique for monitoring hepatic steatosis in an obese population. MRI-derived SAT volume measurements at L2–3 or L4 might be an optimal location for evaluating total body fat mass.

## Data Availability

The dataset used and/or analyzed during the current study are available from the corresponding author on reasonable request.
